# Seasons change and so do trees: Expression profiling of aspen reveals season-specific gene hubs

**DOI:** 10.1093/plcell/koaf213

**Published:** 2025-08-26

**Authors:** Renuka Kolli

**Affiliations:** Assistant Features Editor, The Plant Cell, American Society of Plant Biologists; Sainsbury Laboratory, University of Cambridge, Cambridge, UK

Trees of the *Populus* genus, such as poplar and aspen, are native to the northern hemisphere. These perennials are fast-growing and tolerate a wide range of environmental conditions. Ecologically, they prevent soil erosion and are useful for a variety of microbes and herbivores. Moreover, they are economically important for woodwork, paper manufacturing, and biofuel production. As the species can be grown easily by vegetative propagation, are amenable to transformation, and several genomes have been sequenced, they serve as models for woody perennial plants. Several transcriptomic studies have been completed on *Populus* species grown in controlled conditions to understand stress responses. However, trees in temperate and boreal regions also exhibit substantial seasonal adaptations, including extensive vegetative growth in summer; cold acclimation, leaf senescence, and bud formation in autumn; dormancy in winter; and bud flush in spring.

To study seasonal changes at the molecular level, **Alice Marcon and coauthors (Marcon et al. 2025)** generated RNA sequencing data of leaf or bud samples collected monthly for aspen (*P. tremula*) trees growing outdoors in Sweden, as well as a closely related hybrid (*P. tremula x tremuloides*) grown indoors in controlled conditions mimicking seasonal changes in day-length and temperature. Principal component analysis clustered the samples into 5 groups: autumn and late winter together, winter, April, May, and summer. Interestingly, the May samples were well separated from all other samples. This corresponds to the developmental phase when buds flush and young leaves emerge. Upon including the indoor samples, they roughly grouped with the samples of similar outdoor light and temperature conditions, indicating similar seasonal expression profiles. Coexpression analysis generated 46 modules, of which 36 showed a seasonal profile. The authors selected 12 modules with distinct seasonal expression profiles whose patterns were similar in outdoor and indoor settings. They consist of 2 autumn modules, 2 winter modules, 4 spring modules, and 4 summer modules based on the time of high gene expression (Fig. 1).

**Figure 1.**
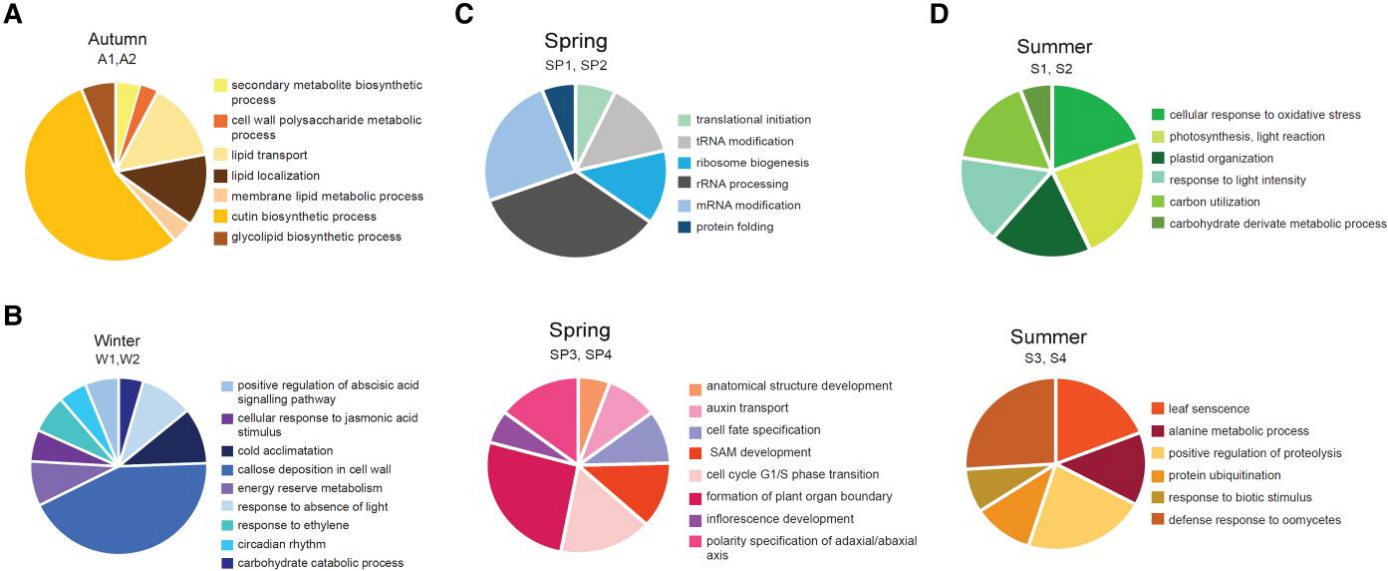
GO enrichment analysis for the gene expression profiles specific to **(A)** autumn, **(B)** winter, **(C)** spring, and **(D)** summer. The module identifiers are written below the season. Adapted from Marcon et al. (2025), Figures 4 to 6.

Gene ontology (GO) term enrichment analysis revealed that most GO terms were specific to each module. During autumn, plant cell membranes and cell walls undergo extensive remodeling to maintain membrane fluidity and increase cell wall thickness to help the plant adapt to lower temperatures and reduced water availability (Takahashi et al. 2021). Accordingly, the identified GO terms include “cutin biosynthetic process,” “lipid localization,” and “cell wall polysaccharide metabolic process” (Fig. 1A). During winter, trees enter a dormant state, and the enriched GO terms concern cold acclimation and response to certain hormones (Fig. 1B). Two spring modules show enrichment in RNA processing and ribosome biogenesis, indicating upregulation of transcription and translation, while 2 other spring modules are enriched in the terms associated with cell cycle and inflorescence development (Fig. 1C). The latter corresponds to the floral transition wherein a few vegetative shoot meristems are converted into inflorescence meristems (Khan et al. 2025). In summer, an active growth period is evident through enrichment of terms related to photosynthesis, and by the end of summer, the enriched terms such as “leaf senescence” and “positive regulation of proteolysis” indicate growth cessation (Fig. 1D).

Marcon et al. (2025) described season-specific gene expression profiles in aspen, created gene subnetworks for each season, and compared transcriptional changes between trees grown outdoors and indoors. Furthermore, they developed an interactive web application called POPUL-R to make the data publicly accessible. POPUL-R provides expression profiles of selected modules along with the gene lists and associated GO terms. It can also be used to explore the expression profile of a single gene of interest or of multiple genes during the annual growth cycle of aspen and obtain their first neighbors in the coexpression network. Thus, this study opens doors to study *Populus* gene regulation during developmental transitions and to characterize candidate proteins at the mechanistic level.

## Recent related articles in *The Plant Cell*

- Xiao et al. (2023) identified PtoP4H9 as a key regulator of stem growth in poplar.
- Gao et al. (2024) identified that PtoHY5a determines the onset of winter dormancy by suppressing short day–induced growth cessation and bud set in poplar.
- Zhang et al. (2024) identified PagMYB31 as a key regulator for wood formation in poplar.
